# History and Profile of Diagnosis Procedure Combination (DPC): Development of a Real Data Collection System for Acute Inpatient Care in Japan

**DOI:** 10.2188/jea.JE20200288

**Published:** 2021-01-05

**Authors:** Kenshi Hayashida, Genki Murakami, Shinya Matsuda, Kiyohide Fushimi

**Affiliations:** 1 Department of Medical Informatics and Management, University Hospital, University of Occupational and Environmental Health, Fukuoka, Japan; 2 Department of Preventive Medicine and Community Health, University of Occupational and Environmental Health, Fukuoka, Japan; 3 Department of Health Policy and Informatics, Tokyo Medical and Dental University Graduate School, Tokyo, Japan

**Keywords:** Diagnosis Procedure Combination (DPC), DPC-based Per-Diem Payment System (DPC/PDPS), patient classification system, health policy, Japan

## Abstract

DPC, which is an acronym for “Diagnosis Procedure Combination,” is a patient classification method developed in Japan for inpatients in the acute phase of illness. It was developed as a measuring tool intended to make acute inpatient care transparent, aiming at standardization of Japanese medical care, as well as evaluation and improvement of its quality. Subsequently, this classification method came to be used in the Japanese medical service reimbursement system for acute inpatient care and appropriate allocation of medical resources. Furthermore, it has recently contributed to the development and maintenance of an appropriate medical care provision system at a regional level, which is accomplished based on DPC data used for patient classification. In this paper, we first provide an overview of DPC. Next, we will look back at over 15 years of DPC history; in particular, we will explore how DPC has been refined to become an appropriate medical service reimbursement system. Finally, we will introduce an outline of DPC-related research, starting with research using DPC data.

## 1. OVERVIEW OF DIAGNOSIS PROCEDURE COMBINATION (DPC)

### 1.1 DPC code

**Figure 1.**
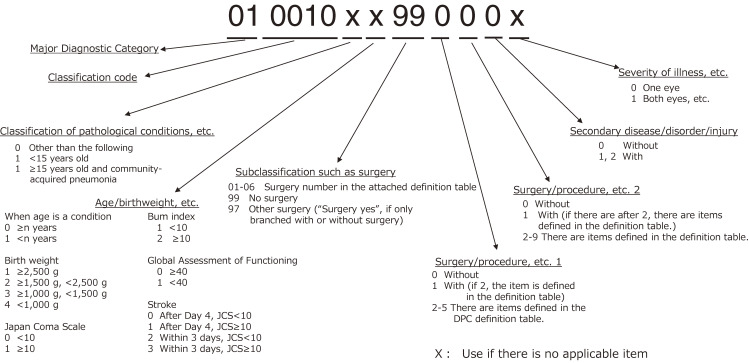
Structure of Diagnosis Procedure Combination codes (as of 2020)

Under DPC, each patient gets a 14-digit code, where each digit has a meaning (Figure 1). The first six digits indicate the diagnosis for which most of the medical resources allocated to a patient were used. It is defined based on ICD-10 codes for each diagnosis. The first two of the six digits indicate a major diagnostic category (MDC). There are currently 18 MDCs. The diagnoses in each MDC corresponds to a single organ system or etiology (Table 1). For example, if the first two digits are “01,” it refers to nervous system diseases and disorders (DDs). The seventh digit formerly indicated “hospitalization type” but now indicates “classification of pathological condition,” such as community-acquired pneumonia. The eighth digit indicates “age and birthweight,” which is used when the amount of medical resources differs depending on patients’ age and (for newborns) birthweight, even if the condition is the same. For example, asthma or pneumonia require a different amount of medical resources in children and adults, and newborns may also require different amounts of medical resources depending on their birthweight. The ninth and tenth digits refer to “type of surgical procedure” prescribed for the DDs indicated by the six digits of each DPC code. The eleventh and twelfth digits indicate adjuvant procedures and therapies: the eleventh digit refers to the adjuvant procedure prescribed in addition to the primary surgical procedure (indicated by the ninth and tenth digits of the DPC code), and the twelfth digit indicates an adjuvant therapy, such as radiotherapy or chemotherapy, that is usually prescribed for the DDs (indicated by the first six digits of the DPC code). The thirteenth digit indicates “comorbidities and complications (CCs),” which include pre-existing comorbidities at the time of admission, sequelae that were directly associated with the surgical procedures or therapies conducted during a patient’s hospital stay, and complications that were not directly related to the surgical procedures or therapies. The fourteenth digit provides additional information that has not been expressed by the preceding thirteen digits, such as factors related to the amount of medical resources required. For example, in the case of cataracts, it presents information about whether one or both eyes are affected. This classification is revised once every 2 years when the system of medical service reimbursement is revised (Table 2).

**Table 1.** Major Diagnostic Categories (MDCs)

MDC code	Description
01	Diseases and Disorders of the Nervous System
02	Diseases and Disorders of the Eye
03	Diseases and Disorders of the Ear, Nose, and Throat
04	Diseases and Disorders of the Respiratory System
05	Diseases and Disorders of the Circulatory System
06	Diseases and Disorders of the Digestive System, Hepatobiliary System, and Pancreas
07	Diseases and Disorders of the Musculoskeletal System and Connective Tissues
08	Diseases and Disorders of the Skin and Subcutaneous Tissue
09	Diseases and Disorders of the Breast
10	Diseases and Disorders of the Endocrine, Nutritional and Metabolic System
11	Diseases and Disorders of the Kidney, Urinary Tract and Male Reproductive System
12	Diseases and Disorders Pertaining to the Female Reproductive System, Pregnancy, Childbirth, and Puerperium
13	Diseases and Disorders of the Blood, Blood Forming Organ and Myeloproliferative Diseases and Disorders
14	Neonatal Diseases and Disorders
15	Pediatric Diseases and Disorders
16	Trauma, Burns, and Poisonings
17	Mental Diseases and Disorders
18	Other Diseases and Disorders

**Table 2.** Changes in the number of DPCs

Revision Date	Number of MDCs	Number of Diseases/injuries	Number of DPCs (Total)	Number of DPCs subject to bundled payment	(Payment categories)
April 2003	16	575	2,552	1,860	(1,860)
April 2004	16	591	3,074	1,726	(1,726)
April 2006	16	516	2,347	1,438	(1,438)
April 2008	18	506	2,451	1,572	(1,572)
April 2010	18	507	2,658	1,880	(1,880)
April 2012	18	516	2,927	2,241	(2,241)
April 2014	18	504	2,873	2,309	(2,309)
April 2016	18	506	4,918	4,244	(2,410)
April 2018	18	505	4,955	4,296	(2,462)
April 2020	18	502	4,557	3,990	(2,260)

DPC, Diagnosis Procedure Combination; MDC, Major Diagnostic Category.

Thus, the DPC combines information on (1) main diagnosis, (2) interventions, and (3) comorbidities/complications and additional information. The process of determining a patient’s DPC code is as follows: 1) When the diagnosis for which most of the medical resources were used is determined, six digits based on the ICD-10 code corresponding to the diagnosis are determined; 2) next, the ninth, tenth, eleventh and twelfth digits of the DPC code are determined by adding necessary information about medical interventions, such as surgical procedures and therapies; 3) finally, the remaining digits of the DPC code are determined by including information about comorbidities, complications, and the severity of the patient’s medical condition. Thus, as DPC code assignment is a process largely consisting of three layers based on diagnosis, it can be argued that DPC is a classification method that emphasizes a patient’s diagnosis and diseases.

### 1.2 Per-Diem Payment System based on DPC

**Figure 2.**
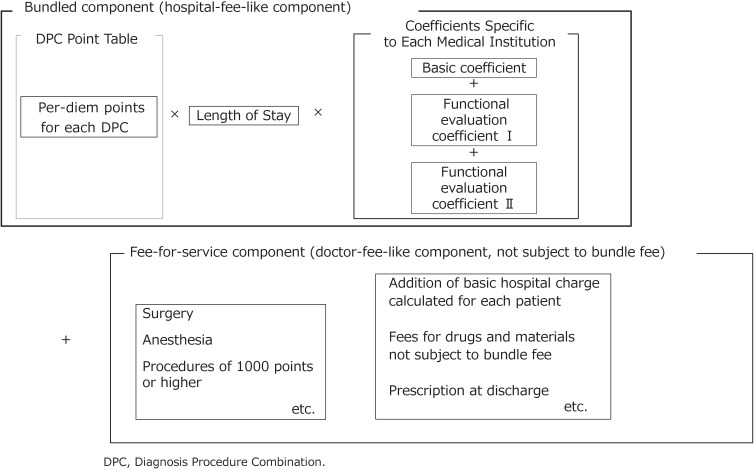
Composition of DPC-based medical service fee payment (bundled component and fee-for-service component)

The DPC-based Per-Diem Payment System (DPC/PDPS) is the main medical service reimbursement system for acute inpatient care in Japan. Part of the charges are calculated based on the bundled payment model (hereinafter “bundled-payment component”) and the other part based on the fee-for-service (FFS) payment model (hereinafter “FFS component”). These are added together to derive the total payment (Figure 2). Patients in general wards, but not rehabilitation or psychiatric wards, are treated under DPC/PDPS. However, the following patients are not paid under the DPC/PDPS but under the FFS system: 1) those whose medical service reimbursement points (average points depending on DPC code) in the bundled-payment component are difficult to calculate because of low number of cases; and 2) those with unique medical conditions. As of April 2020, the payment system initially introduced to 82 hospitals in 2003 has been applied to 1,757 hospitals with a total of 483,180 beds. This number is thought to be enough to cover almost all acute inpatients, and is about 30% of all hospitals with beds for general patients (including those in subacute care and rehabilitation, but excluding those with mental illness, infectious disease, tuberculosis, and long-term care) and about 54% of all beds of hospitals with beds for general patients across the country (Table 3).

**Table 3.** Changes over time in number of hospitals/beds subject to DPC-based Per-Diem Payment System

Fiscal Year	Number of facilities	Number of beds
2003 DPC/PDPS hospitals (as of July 2003)	82	68,982
2004 DPC/PDPS hospitals (as of July 2004)	144	94,115
2006 DPC/PDPS hospitals (as of July 2006)	360	177,806
2008 DPC/PDPS hospitals (as of July 2008)	718	288,282
2009 DPC/PDPS hospitals (as of July 2009)	1,282	433,604
2010 DPC/PDPS hospitals (as of July 2010)	1,390	456,201
2011 DPC/PDPS hospitals (as of April 2011)	1,449	467,511
2012 DPC/PDPS hospitals (as of April 2012)	1,505	479,539
2013 DPC/PDPS hospitals (as of April 2013)	1,496	474,981
2014 DPC/PDPS hospitals (as of April 2014)	1,585	492,206
2015 DPC/PDPS hospitals (as of April 2015)	1,580	484,081
2016 DPC/PDPS hospitals (as of April 2016)	1,667	495,227
2017 DPC/PDPS hospitals (as of April 2017)	1,664	483,747
2018 DPC/PDPS hospitals (as of April 2018)	1,730	488,563
2019 DPC/PDPS hospitals (as of April 2019)	1,727	482,361
2020 DPC/PDPS hospitals (as of April 2020)	1,757	483,180

DPC, Diagnosis Procedure Combination; DPC/PDPS, DPC-based Per-Diem Payment System.

The bundled-payment component of DPC/PPDS is likened to hospital fees (as opposed to physician fees). Medical service reimbursement points in this component are set per day for average medical services, such as examination, prescription, injection, and therapy, by patient category. Unlike in most countries, points are set based on the actual amount of services provided by the hospitals that use the DPC/PDPS. By contrast, the FFS component is likened to physician fees for medical services, such as surgery, anesthesia, radiotherapy, and other procedures that are valued at or over 1,000 points (equivalent to 10,000 yen or roughly somewhat under 100 United States dollars). Therefore, the final amount is calculated as the sum of the bundled payment component and the FFS component. The bundled-payment component is calculated by multiplying per-diem medical service reimbursement points set for the specific DPC by length of stay and an institution-specific coefficient (Figure 2). This is intended to reflect both the difference in the patient’s condition and the difference in the function of the medical institution. That is, the difference in each patient’s condition is reflected by multiplying the per-diem number of medical service reimbursement points by the length of stay, and the difference in the functions of medical institutions is reflected by multiplying the product by the coefficient specific to each medical institution.

**Figure 3.**
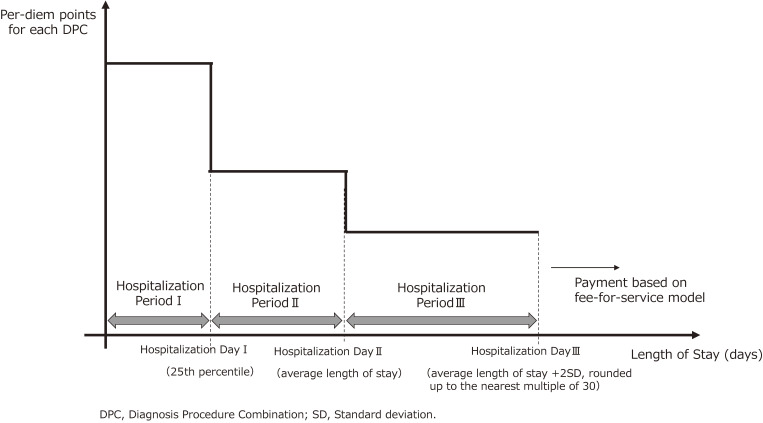
Method of setting the medical service fee points by the length of hospital stay

Per-diem medical service reimbursement points for each DPC gradually decrease, in three stages. This is both because medical resources are used disproportionately in the early stage of a hospital stay and in order to stimulate earlier discharge and streamline medical care. Figure 3 shows the three stages: hospitalization day I corresponds to the 25^th^ percentile for length of stay under the DPC code; hospitalization day II, to average length of stay (ALOS); and hospitalization day III, to the sum of ALOS and two standard deviations (SDs), rounded up to the nearest multiple of 30. For example, if the sum of the ALOS + 2SD is 24, hospitalization day III is 30 days, as the nearest multiple of 30 for 24 is 30. Hospitalization days I, II, and III differ across the DPCs. The medical service reimbursement points are set for each period.

Furthermore, four patterns for gradual decrease of reimbursement points by length of stay are set, to match the pattern of medical resource use: a general or consistent pattern, one using considerable resources at the early stage, one using few resources at the early stage, and one using an extremely large amount of resources, such as high-priced chemotherapy drugs, on the first day of hospitalization (in this case, hospitalization day I corresponds to 1 day rather than the 25^th^ percentile). This is done because a time-series analysis of the patterns of medical resource use among the DPCs was conducted to ensure that they represent actual costs as closely as possible. It is noteworthy that if the length of stay exceeds hospitalization day III, the medical charges, including those for medical services that fall under the bundled-payment component, are calculated based on the FFS system.

Each medical institution has a basic coefficient depending on its function (university hospitals that provide advanced acute care and physician training; DPC-designated hospitals whose functions correspond to those of university hospitals; standard DPC hospitals that provide standard acute care) and two functional evaluation coefficients I and II. Functional evaluation coefficient I evaluates hospital systems (eg, medical record management, medical safety measures, and infection prevention measures), adequacy of nurse staffing, and regional characteristics, such as remoteness or ruralness. Furthermore functional evaluation coefficient II evaluates incentives pertaining to the roles medical institutions should fulfill to improve the efficiency of the entire medical system: how efficiently medical care is provided, how promptly patients are discharged, how many patients with complex medical conditions are treated, how diverse their medical conditions are, and how much the institutions contribute to regional medical care, such as emergency care.

It is noteworthy that this prospective payment system in Japan is characterized by the fact that it does not include a medical cost-containment mechanism. Therefore, when introducing a prospective payment system in 2003, or when setting medical service reimbursement points under the DPC/PDPS at every revision of medical service fees, and introducing and setting coefficients under the DPC/PDPS, only the percentage change in the overall medical service fees was determined while revising the medical service payment, which affected the total amount of disbursements for healthcare financing.

### 1.3 DPC data

Nationally uniform electronic DPC data include patient clinical information and information on medical procedures used for patient classification, and the DPC-based reimbursement system. They are used to improve systems and policies, including for hospital management and service reimbursement. The basic data consists of Form 1, D-file, EF-file, and H-file (Table 4). Form 1 contains information on medical records. The D-file contains information on medical charges based on DPC-based medical service reimbursement points (DPC point table). The EF-file contains information on medical charges based on the FFS system. The H-file contains anonymized information organized by date, obtained from medical records (which currently include points set per evaluation item on the evaluation sheet pertaining to the severity of a patient’s condition and the extent of a patient’s need for medical/nursing care). Furthermore, there are Form 3, Form 4, and the K-file. Form 3 is a facility questionnaire. Form 4 is a questionnaire for patients who receive medical services not covered by public health insurance. The K-file enables coupling analysis for the National Database of Health Insurance Claims and Specific Health Checkups of Japan (NDB) and the Long-Term Care Health Insurance Claims Database (Long-Term Care DB).

**Table 4.** Data related to Diagnosis Procedure Combination (DPC)

File	Description	Item
Form 1	Patient clinical information	Basic patient information (date of birth, sex), postal code of patient address area, dates of admission and discharge, route of hospital admission, outcome at discharge, diseases/injuries name, surgical procedure, various clinical scores, cancer stage classification, etc

D-file	Medical treatment information (Reimbursement information for DPC/PDPS)	Medical charges, implementation date and number of times/quantity of medical treatment (surgery, treatment, test, etc), pharmaceuticals, medical materials, etc

EF-file	Medical treatment information (Reimbursement information for fee-for-service payment)	Medical charges, implementation date and number of times/quantity of medical treatment (surgery, treatment, test, etc), pharmaceuticals, medical materials, etc

H-file	Daily anonymization information from medical records etc	Scores for each evaluation item on the evaluation sheet for severity of a patient’s condition and extent of a patient’s need for medical/nursing care

Form 3	Facility information	Number of beds, medical safety measures, infection prevention measures, etc

Form 4	Information on the patients who have received medical treatment not covered by public health insurance	Payment source information for hospitalization medical charge, etc

K-file	Encrypted file able to conduct coupling analysis of the other database	Primary common ID (generated based on date of birth, name spelled in kana, and sex), date of admission, date of discharge, implementation date of medical treatment, medical institution, etc

DPC/PDPS, DPC-based Per-Diem Payment System.

In more detail, Form 1 contains basic and clinical information about various patients, including date of birth, sex, residential postal code, dates of admission and discharge, route of admission, discharge outcome, diagnosis, surgical procedure, various clinical scores, and stage classification. It can be regarded as a “simplified discharge summary.” The EF-file contains information on medical procedures (type of procedure, date, frequency, administered medication, medical resources, department/ward, and ordering physician). The D-file is prepared by each medical institution that receives medical charges calculated under the DPC/PDPS. The D-file contains points information for the bundled-payment and FFS component, as well as institution-specific coefficients for reimbursement. The H-file includes the value of a specific evaluation sheet assessing the severity of a patient’s condition and the extent of a patient’s need as an index for determining medical treatment fees. It differs by ward type: “general wards,” “intensive care units (ICUs) designated for certain treatment,” and “high-dependency (high care) units.” It contains information on patients’ day-to-day status and medical care provided. Form 3 contains information on each facility, including the number of beds of each department (eg, general, mental, and infectious disease), information related to functional evaluation coefficient I calculation (eg, medical record management, medical safety measures, and infection prevention measures). Form 4 contains information about patients who receive medical services not covered by public health insurance. It also indicates whether a charge was made for treatment provided during hospitalization not covered by public health insurance for each patient. The K-file is an encrypted file that includes a primary common ID automatically generated by a support tool provided by the national Ministry of Health, Labour and Welfare: date of birth, name spelled in Japanese kana characters, and sex, with a code indicating the institution and admission and discharge dates.

In other words, Form 1 and the H-file contain basic and clinical information and the day-to-day status of the patient. The EF-file shows who did what when and to what extent during care. Therefore, the form and files indicate the medical treatment process and the outcomes, that is, it is possible to understand what kind of medical procedures were performed on what type of patients and what the results were. The form and files enable chronological visualization of the process of medical treatment and analysis of the average images and variations as aggregated values. If utilized effectively, DPC data can provide useful information to healthcare professionals, insurers, and policymakers.

## 2. HISTORY OF DPC

DPC started not as a classification method emphasizing diagnosis and diseases, as at present, but as an adaptation of Diagnosis-Related Groups (DRG) classification, developed in the United States. In 1998, the Japanese Ministry of Health (currently the Ministry of Health, Labour and Welfare) introduced per-case payment based on diagnostic group classification on a trial basis in 10 facilities, including national hospitals: the Japanese version of DRG. This trial study aimed to examine whether classifications could be used to provide efficient and high-quality medical services. To this end, changes in the hospitalization length, contents of medical procedures, the level of patient satisfaction, and hospital management from before to after the trial of the classification-based payment system were examined. At that time, experts from each academic society created the classification on the government’s request. In 13 major diagnostic categories there were 270 classifications, of which 183 classifications were paid under the bundled-payment system. In this trial study, hospital costs, medication and material fees, and examination and imaging costs were included in a predetermined, fixed amount by each classification and were paid as a bundle per case. The FFS system was adapted for surgery costs and expensive procedures. As a result, no obvious changes were seen in average length of stay or bed utilization rate from before to after the trial. This seemed to indicate issues with study content and classification method. Furthermore, there were only 183 classifications for payment, covering only 30–40% of inpatients, and some payment amounts had become extremely high for the service. Therefore, the trialed payment method was not adopted.

In 2001, the classification was revised to have 15 major diagnostic categories with 532 classifications (of which 267 were paid under the bundled-payment system). A survey was conducted at 54 private hospitals in order to examine the validity, usefulness, and feasibility of this classification; it involved implementing the classification and surveying participants on the results but not actually making payments. To further improve coverage, a new classification system with a three-layer structure (diagnosis, surgery/procedures, and complications/comorbidities), to fit clinicians’ process and thinking, was developed. The ICD-10 code was used for diagnosis classification, and the k-code, a medical service reimbursement chart that had long been used in the FFS system in Japan, was used for surgery classification. When applying the revised classification to the data collected from special-function hospitals from July to September 2002, the overall applicable rate was 96.32%. This was the first DPC, diagnostic group classification developed in Japan, which was composed of 575 categories by diagnoses (the first six digits), 2,552 categories according to a 14-digit DPC, and 1,860 payment categories.

**Figure 4.**
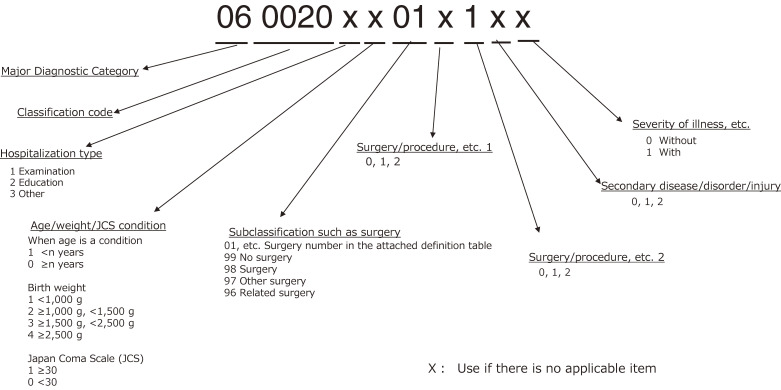
Structure of first Diagnosis Procedure Combination codes (as of Apr 2003)

**Figure 5.**
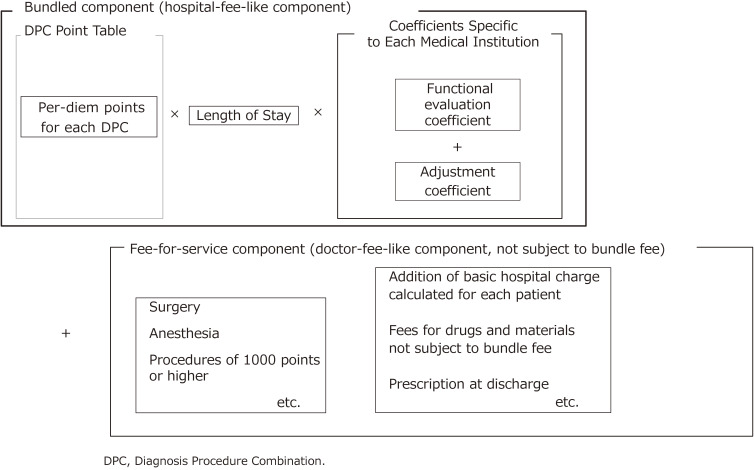
Composition of DPC-based medical service fee payment (as of 2003)

In April 2003, using the first DPC code (Figure 4), developed with the above-mentioned three-layer structure, a per-diem bundled-payment system was started in special-function hospitals. The refinement of the classification was not adequate, and effects on hospital finances from changes in payment method also raised concerns. Therefore, in addition to a functional evaluation coefficient, an adjustment coefficient to ease such effects was introduced (Figure 5). When the per-diem payment system was introduced, there was only one pattern, gradually decreasing dependent on the length of stay.

In the fiscal year (FY) 2004 revision of the medical service payment system, medical service fees were to be paid by the FFS model, not by the bundle component based on the DPC, for cases in which expensive drugs or medical devices were used. As a result, the number of diagnoses and the DPCs increased, while the number of cases eligible for the bundled-payment models decreased (Table 2). In addition, for DPCs with a large amount of medical resources input early during hospitalization, such as chemotherapy for malignant tumors, a new setting method was to be used for medical service fee reimbursement points based on the length of hospital stay. With this change, there were two decreasing patterns which were dependent on the length of hospital stay. The new pattern shortened the initial period of hospitalization (decreased from the 25^th^ percentile to the 5^th^ percentile of days of hospital stay for each DPC) and increased the points during that period. From 2004, the application of the DPC system to medical facilities was expanded to a broader range of hospitals, including private hospitals, and was no longer limited to the special-function hospitals, in which the system was already used (Table 3).

In the FY 2006, in order to revise the medical service payment system and to refine DPC, the following were implemented: (1) revision of surgery and surgery/procedures, (2) cancellation of the use of hospitalization types (hospitalization for examinations; educational hospitalization [ie, hospitalization to promote patient education, such as lifestyle improvement]), in the seventh digit, and (3) revision of secondary diseases. The major changes for each of the above were as follows. (1) The classification by surgery grouped similar surgeries for each MDC. In addition, definitions of surgeries/procedures that varied even for the same disease were set. (2) Due to the ambiguous definitions, categorization by hospitalization types in the seventh digit was no longer to be used; instead, a classification method based on specific medical procedures, such as examination and treatment, was to be used. (3) Disease names with vague definitions were excluded. As a result, the numbers of included disease/injury names and DPCs became 516 and 2,347, respectively (Table 2).

In the FY 2008 revision of the medical service payment system, the rules for medical service fee calculations were revised and the DPC was refined. The major revisions of the DPC included reorganization of MDCs for certain diseases and the number of MDCs was increased from 16 to 18. MDC16 was previously a group of diseases with completely different clinical concepts (eg, trauma/burns/poisoning, mental illness). Diseases caused by extrinsic factors, such as trauma, burns, and poisoning, were kept in MDC16; mental disorders were moved to MDC17; and the remaining were included in MDC18 (Table 1 and Table 2). Simultaneously, AIDS and hemophilia were moved from MDC16 to MDC13. In addition, the disease/injury category was reviewed to reflect the major standard regimens of cancer chemotherapy in the classification. After the review, the numbers of diseases and injuries were 506, DPCs 2,451, and bundled-payment categories 1,572 (Table 2). In addition, the medical service fee calculation rules were revised to treat readmission within 3 days after discharge as one hospitalization.

In the FY 2010 revision of the medical service payment system, major changes were made and new functional evaluation coefficients were introduced. As described above, when the DPC/PDPS was first introduced, the coefficient specific to each medical institution consisted of a functional evaluation coefficient and adjustment coefficients; the latter were aimed to secure “a similar level of income as in the previous fiscal year,” to alleviate sudden changes in the financial situation of the institution due to the changes in the fee payment method. However, because refinement of the classification advanced, a new coefficient to evaluate hospital functions was created to abolish the adjustment coefficient, which was not covered by the previous functional evaluation coefficient. The new functional evaluation coefficient was supposed to reflect the “acute phase” and was able to evaluate transparency, efficacy, standardization, and quality improvement. Furthermore, the functions and roles expected by society as a DPC hospital were emphasized, and contribution to regional medical care was also deemed necessary. The previous functional evaluation coefficient was renamed functional evaluation coefficient I. The adjustment coefficient was replaced with a new functional evaluation coefficient, in a stepwise manner. First, it was partially (25%) replaced with functional evaluation coefficient II, consisting of six elements: data submission index, efficiency index, complexity index, coverage index, regional medical care index, and emergency medical care index. The revision of the rules of calculation of medical service fee points included the following: The decreasing pattern with a short initial hospitalization period that was introduced in FY 2004 was abolished, and the initial stage of hospitalization was fixed at 25^th^ percentile of the length of the hospital stay for each DPC. However, for DPCs with a large amount of medical resources input in the initial hospitalization period, medical service fee points were set high for the initial period, while for DPCs with a small amount of medical resources input in the initial hospitalization period, low points were set. The decreasing patterns set were three (ie, from FY 2004 there were two types of decreasing patterns, which were revised in FY 2010 when one pattern was abolished and two more were introduced). For the refinement of the DPC, the use of high-priced drugs and chemotherapy regimens were reflected in the classification, and the secondary diseases that were previously set for each 6-digit DPC classification were further differentiated according to whether surgery was performed.

The FY 2012 revision of the medical service payment system included the following major changes: (1) introduction of a basic coefficient and setting of medical institution groups, (2) replacement of adjustment coefficient and revision of functional evaluation coefficient, and (3) changes in the comprehensive evaluation of high-priced drugs. The main points of each change are as follows: (1) A basic coefficient was set for each medical institution group, categorized by focusing on role and functions and classified into the following three groups: Group I DPC hospitals, Group II DPC hospitals, and Group III DPC hospitals. (2) The adjustment coefficients, equivalent to 25% of what they had been before the introduction of functional evaluation coefficient II in 2010, were replaced with the basic coefficient and the new functional evaluation coefficient II. In addition, the items and the calculation methods of functional evaluation coefficient I and II were revised. (3) In hospitalization cases for chemotherapy for cancer patients, the input of resources such as high-priced drugs occurs on the first day. Therefore, the initial hospitalization period is set to 1 day, and there is a new payment pattern by which the medical service fee points for the first day are set higher than usual.

In the revision of the FY 2014 medical service payment system, major revisions were made in the following: (1) response to the consumption tax rate increase, (2) revision of the coefficient specific to each medical institution, and (3) revision of the definition of rehospitalization and prohibition of the use of medications brought into the hospital by a patient.

The main points of each change are as follows: (1) the medical service fee points were changed to reflect the increase in the consumption tax rate from 5% to 8%; (2) 50% of the previous adjustment coefficients were replaced with functional evaluation coefficient II, and the rest of the adjustment coefficient was set as the provisional adjustment coefficient (the “generic drug index,” which evaluates the use of generic drugs in inpatient medical care, was added to functional evaluation coefficient II, for a total of seven elements); and (3) the definition of re-hospitalization as one continuous hospitalization, which previously referred to readmission within 3 days for the same disease, was revised as follows: readmission within 7 days for the disease/injury of the same MDC as the disease/injury for which the largest input of medical resources was made during the previous hospitalization. In addition, in principle, the revision prohibited prospective inpatients from using medications while hospitalized that they had brought into the hospital with them for the disease/injury which was the cause of hospitalization. As a result of these revisions, the number of MDCs became 18, diseases/injuries 504, DPCs 2,873, and categories subject to bundled payment 2,309 (Table 3).

In the FY 2016 revision of the medical service payment system, the following actions were made: (1) revision of coefficient specific to each medical institution, (2) revision of the method of setting the end date in the bundled-payment system, (3) revision of the method of setting the bundled-payment classifications, and (4) submission of daily patient status data. The main points of each change are as follows. (1) A total of 75% of the previous adjustment coefficients were replaced with functional evaluation coefficient II. In addition to the evaluation of surgical technology, which had already been included, evaluation of internal medicine technology was newly added to the medical service performance requirement as one of the criteria for being judged a group II DPC hospital (a high-functioning hospital group). A “severity index,” which evaluates the rate of divergence of patient’s severity, had not been previously covered by the DPC, and was newly added to functional evaluation coefficient II. As a result of these revisions, functional evaluation coefficient II consisted of eight elements. (2) Hospitalization day III, which is the end date of the bundled-payment system, was extended to a value that is sum of ALOS and two SDs, rounded up to the nearest multiple of 30. (3) The Comorbidity Complication Procedure Matrix, an evaluation method that considers the severity in the DPC point table, was introduced for three diseases: cerebral infarction, pneumonia, and diabetes. There was a limitation with the DPC structure that is based mostly on disease/injuries that require the highest number of medical resources, in setting classifications that reflect the degree of the medical resource needs in detail without unnecessarily increasing the number of classifications. Therefore, this evaluation method uses a matrix to express the degree of need for medical resources. (4) Data on the severity of the condition and the degree of the medical/nursing care needs were to be collected.

In the FY 2018 revision of the medical service payment system, the main revisions were related to coefficients specific to each medical institution. Specifically, the replacement of the adjustment coefficient with functional evaluation coefficient II, which was implemented from the FY 2012 revision, was almost complete. In addition, the names of medical institution categories (group I–III DPC hospitals) were changed to the university main hospital group, DPC-designated hospital group, and DPC-standard hospital group. In functional evaluation coefficient II, only the six coefficients (medical services under health insurance, efficiency, coverage, complexity, emergency medical care, and regional medical care) that existed at the time of initial introduction were positioned as basic evaluation axes; the two coefficients (generic drugs and severity) that had been added during the revision process were abolished.

In the FY 2020 revision of the medical service payment system, when transferring from a general ward that is subject to DPC/PDPS to a community comprehensive care ward/room, the medical fee payments changed from being based on fee points of community comprehensive care to being based on those of DPC/PDPS until hospitalization day II.

As described above, DPC has been revised based on data, taking into consideration the current situation and prospects of medical care in Japan. DPC, which aims to improve the quality of medicine, has been refined while playing a major role in the development of the medical service fee system.

## 3. DPC-RELATED STUDIES

To date, various DPC-related studies have been conducted. Matsuda, who played a leading role in the development of DPC, stated that the aim of introducing DPC was not only to develop a payment system for medical fees but also to modernize the medical system, mainly in terms of improvement of the quality of hospital management, strengthening the responsibility of hospitals for accountability, and streamlining medical care systems.^1^ Fushimi et al stated that DPC, which is a case-mix system that prioritizes diagnoses, has been used to evaluate the function of health care service providers and that DPC can evaluate services for in- and outpatients comprehensively and can be used to appropriate distribution of medical care resources among health care providers.^2^ In other words, studies that lead to quality improvement of the following are feasible: medical care in the clinical setting, hospital management (especially business aspects), and health care systems and policies. In addition, DPC data include various patient information, such as sex, age, and disease/injury names, and therefore, can be used as detailed statistical information on diseases. Based on these points, we introduce herein some of the major types of studies that use DPC data: (1) studies on diseases and their treatment methods, prognosis, and information about medical resource consumption, using a descriptive epidemiological approach; (2) studies that contribute to quality improvement of medical care in clinical settings; (3) studies that contribute to quality improvement of hospital management; (4) studies that contribute to quality improvement of health care systems and policies; and (5) other studies.

First, there are some studies on the patient background, treatment methods, and outcomes of diseases using a descriptive epidemiological approach. Yasunaga et al demonstrated that inpatients with severe measles show two age-related peaks of onset and clarified the incidence ratio of each measles-related complication.^3^ Yasunaga et al also studied the incidence, sex, and mean age of inpatients with bowel anisakiasis, the ratio of laparotomy and laparoscopic surgery as surgical procedures, and the length of hospital stay as the outcomes.^4^ Yamaoka et al conducted a study on abusive head trauma in children younger than 12 months and reported the incidence and peak age (in months) of onset.^5^ Sasabuchi et al conducted a study on patients who underwent epidural analgesia for acute pancreatitis and showed patient background, inpatient mortality, and rate of complications.^6^ Kunisawa et al classified venous thrombosis into deep vein thrombosis (DVT) and pulmonary thrombosis (PT) and showed that the incidence of DVT and PT was 0.19% and 0.05%, respectively, demonstrating that DPC data-based epidemiological surveys are useful.^7^ Yoshimoto et al used DPC data from 370 hospitals participating in the Japanese Neurosurgical Society Training Program and showed the data from patients with a malignant brain tumor: breakdown of treatment methods performed, including surgery, chemotherapy, radiotherapy, and combination therapy; implementation status of chemotherapy regimen patterns; length of hospital stay; and medical care costs.^8^

There are also a lot of studies that contribute to the improvement of quality of medical care in clinical settings. Some studies have analyzed the association between patient attributes and treatment methods and other have analyzed patient attributes and treatment methods and their effect on patient outcomes. For example, Kobori et al demonstrated that inpatients with Guillain–Barré syndrome who also had cytomegaloviral disease and herpes simplex infections at the same time needed a ventilator significantly more often.^9^ Hayashida et al clarified the usage pattern of sedatives in patients on a ventilator during their ICU stay, and analyzed how difference in patterns affected in-hospital mortality rate and the duration of the use of an artificial ventilator.^10^ Masuda et al confirmed the incidence of pulmonary embolism after spinal surgery and the associated factors. They reported that the incidence of pulmonary embolism increased with advanced age, longer anesthesia, and spinal trauma.^11^ Similarly, Shoda et al conducted a study on factors of in-hospital mortality in patients with hip fracture.^12^ Fujii et al focused on infectious disease as a factor that extends the hospital stay.^13^ Ito et al studied the factors that increase the cost of hospital medical care for patients with colonic diverticular bleeding.^14^ Abe et al examined the factors related to the length of hospital stay and in-hospital mortality in elderly pneumonia patients with dementia.^15^ Morishima et al used the Charlson Comorbidity Index to study on the effects of comorbidities on survival rates of gastric, colorectal, and lung cancers.^16^ Iwashita et al clarified the differences in the duration of the use of a mechanical ventilator, mortality rate, and intensive care treatment implementation status depending on admission to an intensive care unit.^17^ Kuwabara et al investigated the relationship between the use of albumin and catecholamines for subarachnoid hemorrhage and in-hospital mortality, consciousness deterioration at discharge, and reintubation rate.^18^ Kido et al conducted a comparative study on the differences in in-hospital mortality in patients with acute lung injury and acute respiratory distress syndrome based on the administration of sivelestat.^19^ Umegaki et al conducted a comparative study on in-hospital mortality between surgical aortic valve replacement and transcatheter aortic valve implantation for aortic valve stenosis.^20^ Fujimoto et al conducted a study on the association between the status of implementation of rehabilitation before and after surgery and the occurrence of postoperative pulmonary complication in surgically treated lung cancer patients.^21^ Studies have also been conducted on factors that are thought to have a particularly large impact on treatment performance and patient outcomes, such as the introduction of guidelines and new technologies, facility characteristics (eg, number of hospital beds, number of cases in which a specific medical procedure is conducted), and socioeconomic factors. Shirai et al surveyed the selection of chemotherapy regimens and the dose of the drugs used for the treatment of ovarian cancer, focusing on compliance with the treatment guidelines.^22^ Horiguchi et al focused on the emergence of a drug-eluting stent, which was a new technology at the time. They compared DPC data before and after the emergence of a drug-eluting stent to clarify its impact on the implementation rates of percutaneous coronary intervention and coronary artery bypass grafting for angina pectoris and acute myocardial infarction patients.^23^ Regarding the effects of facility characteristics, Murata et al clarified if admission to a teaching or non-teaching hospital had an effect on the risk-adjusted length of hospital stay and in-hospital mortality in patients with bleeding peptic ulcers.^24^ Kaneko et al demonstrated the association between outcomes of surgical procedures, such as occurrences of dislocation and infection, and the surgical volume at the facility in total hip arthroplasty patients.^25^ Tomioka et al showed that there was no association between the socioeconomic status of the patient’s residential area and the determination of treatment policy in patients with hip fracture.^26^ Thus, many studies based on DPC data have contributed to the improvement of medical care in clinical settings, and they have been able to do so due to the advantage that DPC is classified from mainly the clinical viewpoint not the payment viewpoint and DPC data contains detailed information of medical procedures.

A few studies have also been conducted with DPC data that contribute to the improvement of hospital management and health care systems/policies quality, although the number is not very high. One such study is by Tanaka et al, who conducted a study to develop an index that evaluates the efficiency of operating room management, taking facility size and workforce into consideration.^27^ Some studies have examined the effect of introducing a “regional inter-provider care planning fee” into the medical fee schedule as an effect of health policy change. This promotes the implementation of regional clinical pathways aiming to standardize and optimize medical care through collaboration between multiple facilities in a region. Fujino et al reported that the length of a hospital stay decreased at individual and facility level for stroke patients.^28^ Mine et al also reported that the hospital stay of hip fracture patients after surgery shortened.^29^ To evaluate the introduction of DPC/PDPS, Hamada et al conducted a study to clarify the length of stay, total accumulated medical charges, in-hospital mortality, and readmission rate before and after the implementation of DPC/PDPS in acute myocardial infarction patients.^30^

In addition, studies that apply and develop DPC data have also been conducted. Fukuda not only used DPC data but also linked it with other data, such as those on the occurrence of nosocomial infection collected by the Japan Nosocomial Infectious Surveillance by the Ministry of Health, Labour and Welfare. Then, he investigated the factors affecting the occurrence of surgical site infection in gastrointestinal surgery.^31^ Lee et al developed a method of identifying the occurrence of healthcare-associated infections based on the use pattern of antibiotics, which can be grasped from DPC data, and verified this method in medical records.^32^

Based on the above, DPC data have great potential and can be used in studies on health service and policy evaluation. Although DPC data do not contain detailed clinical information, such as test results, they can be used in an even wider range of research in the future.
